# 24-month HIV-free survival among infants born to HIV-positive women enrolled in Option B+ program in Kigali, Rwanda: The Kabeho Study

**DOI:** 10.1097/MD.0000000000009445

**Published:** 2017-12-22

**Authors:** Michelle M. Gill, Heather J. Hoffman, Dieudonne Ndatimana, Placidie Mugwaneza, Laura Guay, Gilles F. Ndayisaba, Emily A. Bobrow, Anita Asiimwe, Lynne M. Mofenson

**Affiliations:** a Elizabeth Glaser Pediatric AIDS Foundation; b Department of Epidemiology and Biostatistics, Milken Institute School of Public Health, The George Washington University, Washington, DC; c Rwanda Biomedical Center/Ministry of Health; d Rwanda University Teaching Hospitals (CHU), Kigali, Rwanda.

**Keywords:** HIV-free survival, lifelong antiretroviral therapy, option B+, prevention of mother-to-child transmission, Rwanda

## Abstract

Lifelong antiretroviral therapy (ART) provision to all pregnant HIV-positive women (“Option B+”) has been recommended by the World Health Organization since 2013, but there remain limited data on the effects of Option B+ on long-term HIV-free survival in breastfeeding HIV-exposed infants. The Kigali Antiretroviral and Breastfeeding Assessment for the Elimination of HIV (Kabeho) study enrolled HIV-positive women from the third trimester of pregnancy to 2 weeks postpartum in 14 heath facilities implementing Option B+ in Kigali, Rwanda. Mother–child pairs in the longitudinal observational cohort were followed until 24 months postpartum, with HIV diagnostic testing at 6 weeks, and 9, 18 and 24 months. The Kaplan–Meier method was used to estimate HIV transmission, survival, and HIV-free survival through 24 months. We enrolled 608 HIV-positive women in 2013–2014; birth outcome data were available for 600 women and 597 live-born infants. By 6 weeks, 11 infants had died and 3 infants had confirmed HIV infection (0.5% transmission; 95% confidence interval [CI] 0.2–1.6). At 9 months, there were 9 additional deaths and 2 new infections (cumulative transmission 0.9%, 95% CI 0.4–2.2). At 18 months, there were 6 additional deaths and no new infant infections. At 24 months, there were no additional child deaths and 1 new infection (cumulative 2.2%, 95% CI 0.7–7.0), for an overall 24-month HIV-free survival of 93.2% (95% CI 89.5–95.6). Low transmission rates and high HIV-free survival at 24 months were achieved in breastfeeding infants of HIV-positive mothers receiving universal ART in urban health facilities in Rwanda, though vigilance on maintaining viral suppression for ART-experienced women is needed.

## 1 Introduction

Elimination of mother-to-child HIV transmission is a global public health priority.^[1]^ In 2013, the World Health Organization (WHO) recommended antiretroviral therapy (ART) administration to all HIV-positive women during pregnancy and breastfeeding, regardless of CD4 cell count or clinical stage, for prevention of mother-to-child transmission (PMTCT), with ART duration through at least cessation of breastfeeding. In generalized HIV epidemic settings, ART was to be given for life (referred to as “Option B+”).^[2]^ This was broadened in 2015 to recommend lifelong ART for all HIV-positive individuals, including pregnant/breastfeeding women, regardless of clinical/immune status.^[3]^ Although there are promising results on program effectiveness for prevention of early infant HIV transmission, there is a paucity of data on the long-term effectiveness of these guidelines through the duration of breastfeeding and on 24-month HIV-free survival.

In April 2012, the Government of Rwanda began national implementation of Option B+ as well as an innovative infant feeding counseling and support program with modules specifically focused on optimizing PMTCT for HIV-positive women. This was part of the Rwanda National Strategy for the Elimination of Mother-to-Child Transmission (eMTCT), with a target to reduce HIV transmission to 2% at 18 months postpartum by 2015.^[4]^

The Kigali Antiretroviral and Breastfeeding Assessment for the Elimination of HIV (Kabeho) study was a longitudinal observational study conducted between 2013 and 2016 among HIV-positive women participating in the national PMTCT program in Kigali, Rwanda, that was designed to evaluate the effectiveness of the Rwandan eMTCT program, including evaluation of 24-month HIV-free infant survival. Further details on enrollment of the cohort are provided elsewhere.^[5]^

## 2 Methods

Between April 2013 and January 2014, 608 HIV-positive women were enrolled from 14 high-volume (defined as caring for >50 HIV-positive pregnant women/year) health facilities in Kigali. Women were eligible if they had documented HIV infection, were in the third trimester of pregnancy or within 2 weeks postdelivery, enrolled in the PMTCT program during antenatal care at 1 of the study sites, planned to remain in the Kigali area after delivery, and were able and willing to provide informed consent for herself and her infant. All women were expected to have initiated lifelong ART for this pregnancy if they were not already on treatment (in line with Option B+ national policy); however, being on treatment was not a criterion for study inclusion. Per ethical guidelines in Rwanda, informed consent was also required from parents of unmarried pregnant women aged less than 18 years. The final 24-month study visits were completed in May 2016.

Women and their infants were followed at monthly clinic visits that coincided with follow-up visits required for the national PMTCT program. During study visits, demographics, HIV and ART-related history, ART adherence (assessed by self-reported recall of doses missed in the past 3 days), ART side effects, and nutritional and infant feeding information were collected. Medical and laboratory information were also abstracted from the relevant facility records. Data were reviewed for completeness and quality, and entered in the study database using SurveyCTO Version 2.10 (Dobility, Cambridge, MA). All study staff received training on the protocol, data collection and the protection of human subjects in research.

HIV infection status was determined from routine test results performed using national procedures and algorithms for infant HIV diagnosis: HIV DNA polymerase chain reaction (PCR) at 6 weeks, and initial rapid HIV antibody testing at age 9, 18, and 24 months, followed by a confirmatory HIV DNA PCR test if antibody positive. Children with an initial HIV DNA PCR positive test result had a confirmatory PCR performed; HIV infection was diagnosed in infants with a confirmed positive PCR assay. Additionally, a dried blood spot (DBS) specimen was taken at the birth visit to retrospectively determine the infants’ HIV status at birth. Children diagnosed with HIV infection were referred to HIV care and treatment services. Maternal viral load (VL) was evaluated by HIV RNA PCR at the time of enrollment and within 2 weeks of delivery, at 18 and 24 months. PCR testing for VL (HIV RNA) and early infant diagnosis (HIV DNA) were conducted at the Rwanda National Reference Laboratory using Roche COBAS Ampliprep/COBAS TaqMan HIV-1 test quantitative test (v2.0) and Roche COBAS Ampliprep/COBAS TaqMan HIV-1 qualitative test (v2.0), respectively.

Ethical approvals were obtained from the Rwandan National Ethics Committee, the Rwanda National Health Research Committee, and the George Washington University Institutional Review Board. Participants provided written informed consent prior to the conduct of study procedures. The protocol is registered at clinicaltrials.gov (NCT02295800).

### 2.1 Statistical analysis

A sample size of 608 permitted an estimate of the HIV-free survival at 18 and 24 months achieving a 3% precision level. This was based on the assumption that 91.9% of infants would be alive and HIV-free at 18 months, using an expected 3% HIV infection rate and an infant mortality rate in Kigali of 55 per 1000 live births, with a 2-sided 95% confidence interval (CI) around the observed proportion and a design effect of 1.6.^[6,7]^

Descriptive summary statistics were calculated for predictor and outcome variables of interest using frequencies and percentages for categorical variables. Medians and interquartile ranges (IQRs) are presented for continuous variables. Select demographic and clinical characteristics were presented separately for the women whose children became HIV-infected during the study period. Undetectable HIV VL is defined as <20 copies/mL, which was the lower limit of detection of the laboratory assay. Kaplan–Meier curves were used to graphically represent mortality, transmission and the combined effect of HIV-free survival up to 24 months. Transmission data for children were censored at the date of their first HIV-positive blood test. All statistical tests were 2-sided and the level of statistical significance was set at 0.05. Statistical analyses were conducted using SAS 9.4 (Cary, NC).

## 3 Results

**Figure 1:**
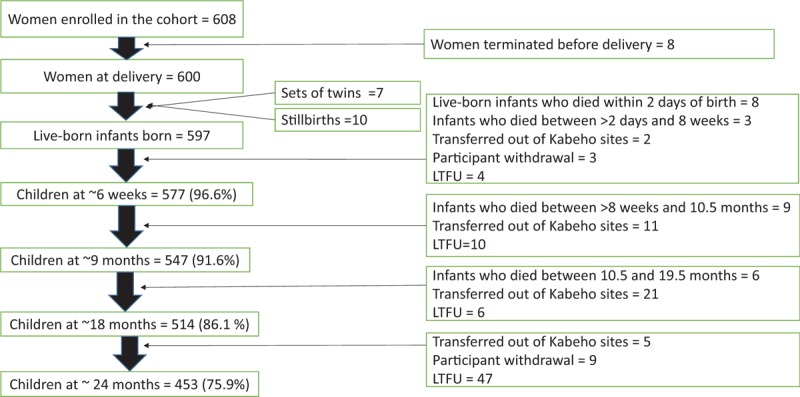
Follow-up of Kabeho study infants. Children remaining in the cohort following delivery and through 24 months of age at HIV testing time points of 6 weeks, 9, 18, and 24 months. Children who died, transferred out of study facilities or whose caregivers withdrew their consent were terminated. Other infants who were expected at these time points but did not return for any further study visits were considered lost to follow-up (LTFU).

Of 721 eligible women, 608 women were enrolled in the Kabeho study, 84.8% during the third trimester of pregnancy and 15.2% within 2 weeks postpartum. Birth outcome data were available for 600 HIV-positive women and their 597 live-born infants, including 7 sets of twins; there were 10 stillbirths (1.6%) (Fig. 1). At the end of the 2-year postpartum follow-up period, retention among children in the study was 75.9% (n = 453). Out of 37 women who had a study visit around 18 months, but did not attend their 24-month study visit, 22 women did come to the facility for their routine clinic visit at approximately 24 months. Primary reasons for loss to follow-up after delivery were transfers from Kabeho sites to nonstudy sites for clinical care (n = 39) and participant withdrawal (n = 20).

Median age was 29.0 years; most women (79.0%) were married/cohabiting (Table 1). Educational attainment was low: 16.2% of women had no education and 60.3% attended primary school only. Only 2.2% had a home with private toilet and 6.7% had piped water. Nearly three-quarters of study women (73.5%) knew their HIV status prior to the current pregnancy, for a median time of 65.4 months (IQR 32.5, 99.5). Only 26.5% of women were first diagnosed with HIV during the current pregnancy. Disclosure of HIV status to a partner was common; 81.7% of women had disclosed to their partner, and 82.2% knew the HIV status of the father (50.3% HIV-positive, 31.9% HIV-uninfected, and 17.8% of unknown status). Median ART duration was 13.8 months; 63.5% of women (n = 381) were already on ART at their first ANC visit. The most common ART regimen was tenofovir/lamivudine/efavirenz, corresponding with Rwandan policy at the time. Although all study participants were enrolled in the PMTCT program, 14 women were not on ART at study entry, 3 of whom enrolled postpartum. Reasons for nonreceipt of ART included provider postponing ART initiation until serum creatinine test results were returned (n = 4), women who had previously been lost to follow-up (n = 4), drug stock-out (n = 1), refusal to take ART (n = 1), interrupted treatment (n = 1), and not yet initiated without specific reason cited (n = 3).

**Table 1:**
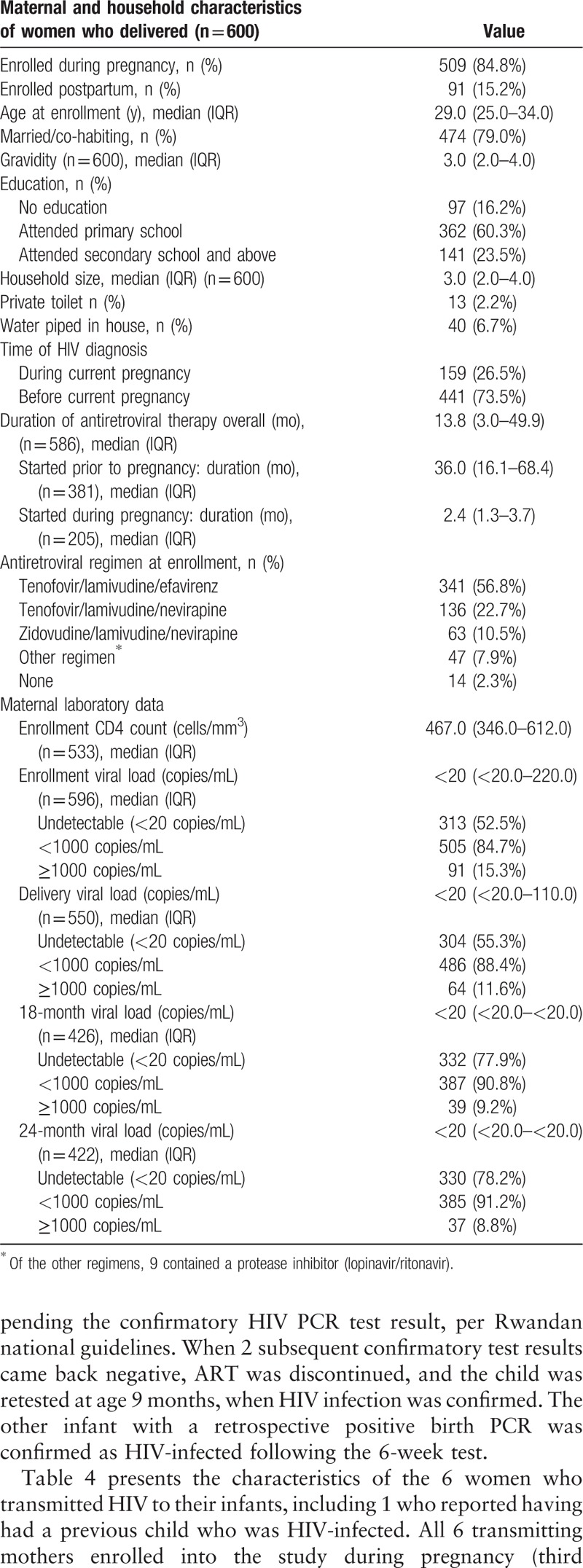
Characteristics of women enrolled in the Kabeho study.

At enrollment, 52.5% had undetectable plasma VL (<20 copies/mL) and 84.7% had HIV RNA <1000 copies/mL (Table 1). Similar to the enrollment results, 55.3% had undetectable plasma VL and 88.4% had HIV RNA <1000 copies/mL at delivery. 24-month VL results were only available for 70.3% of study women who had delivered. Of these, 78.2% had undetectable plasma VL and 91.2% had HIV RNA <1000 copies/mL.

Thirty-six women reported having had a previous child who was HIV-infected with the most recent transmissions occurring in 2012; 3 of these women had 2 previous HIV-infected children. The majority of these previous infections (25/39) occurred when women were not yet aware of their HIV-positive status. Only 1 was on lifelong ART prior to the index pregnancy and 4 were started on ART for life during that pregnancy. Of the 35 women with VL data available around the time of delivery of the study infant, 31 women had a VL of <1000 copies/mL. One of these women transmitted to a study infant; she had detectable viral load at all study measurements.

### 3.1 Pregnancy outcome and maternal and infant mortality

Most women had a vaginal delivery (80.5%) and delivered at a health facility (96.5%). Maternal complications at delivery were reported for 13.8% of women (n = 83), with the most common being prolonged labor (n = 38), maternal hemorrhage (n = 10), fetal distress (n = 9), and breech delivery (n = 7). Overall, there were 3 maternal deaths, for a 24-month maternal mortality rate of 0.5%; 1 woman died after giving birth, 1 died a week after giving birth, and 1 died when her child was over age 18 months.

There were 10 stillbirths (1.6%). Of the 597 live births, 51.8% were female (Table 2). Birth defects were identified in 1.3% infants (n = 8): 3 with polydactyly, 3 lingual frenulum, 1 imperforate anus, and 1 congenital heart disease. Only 1.3% infants (n = 8) were preterm (<37 weeks gestation), and 5.6% (n = 33) had a birth weight below 2500 grams, as measured within 7 days of birth. Almost all infants received 6-week nevirapine prophylaxis (96.4%), with 80.1% initiating nevirapine immediately after birth. Of the 589 infants surviving more than 2 days after birth, most (97.6%) were breastfed; exclusive breastfeeding was reported by 88.0% at age 1 month and 61.2% at 5 to 6 months.

**Table 2:**
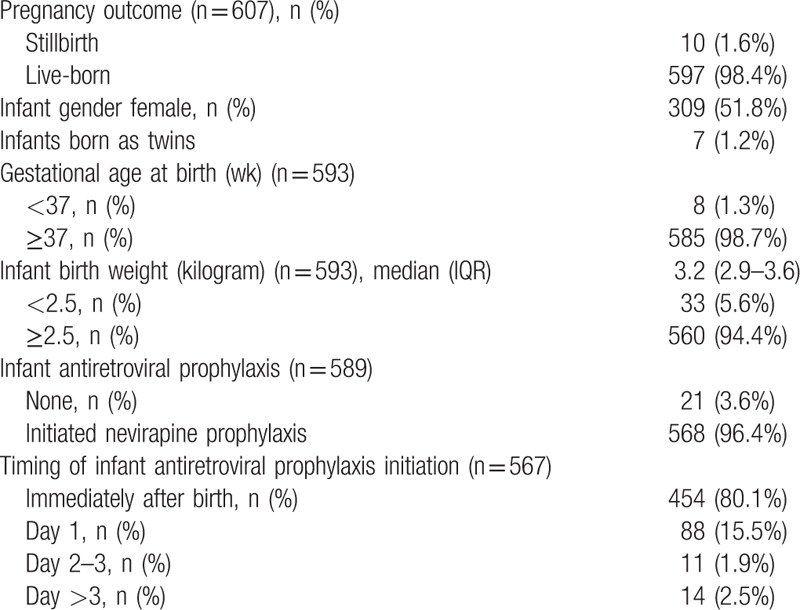
Characteristics of infants enrolled in the Kabeho study.

### 3.2 Infant mortality

**Figure 2:**
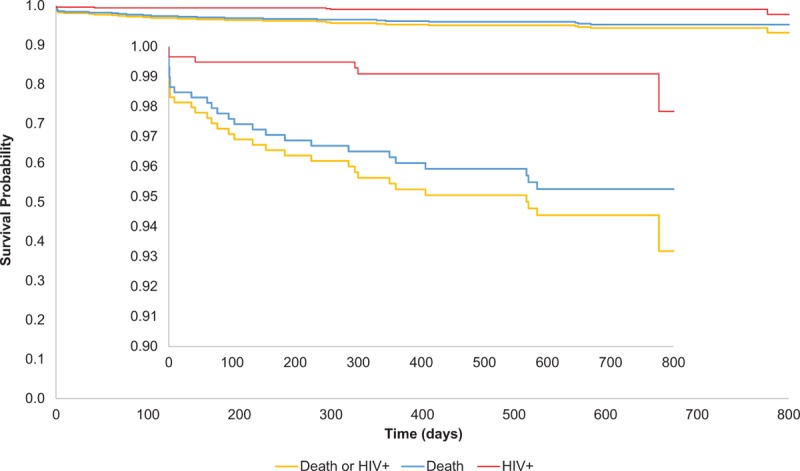
Kaplan–Meier cumulative probabilities of child mortality (blue), HIV transmission (red) and HIV-free survival (yellow) up to age 24 months.

The Kaplan–Meier cumulative probability of child mortality is shown in Figure 2 and Table 3. There were 26 child deaths, including 3 infants who died within 2 hours of birth and 5 others within 2 days. Estimated 24-month mortality was 4.8% (95% CI 3.3–6.9). Reported causes of infant mortality included diarrhea (n = 1), respiratory infection (n = 3), tuberculosis (n = 1), other infections (n = 2), birth asphyxia (n = 2), anemia (n = 1), fever (n = 1), and malnutrition (n = 1). Causes of child death were unknown for 14 children. Data on HIV infection status at last visit prior to death or birth DBS was available for 65.4% (n = 17) live born infants who died; none were found to be HIV-infected.

**Table 3:**
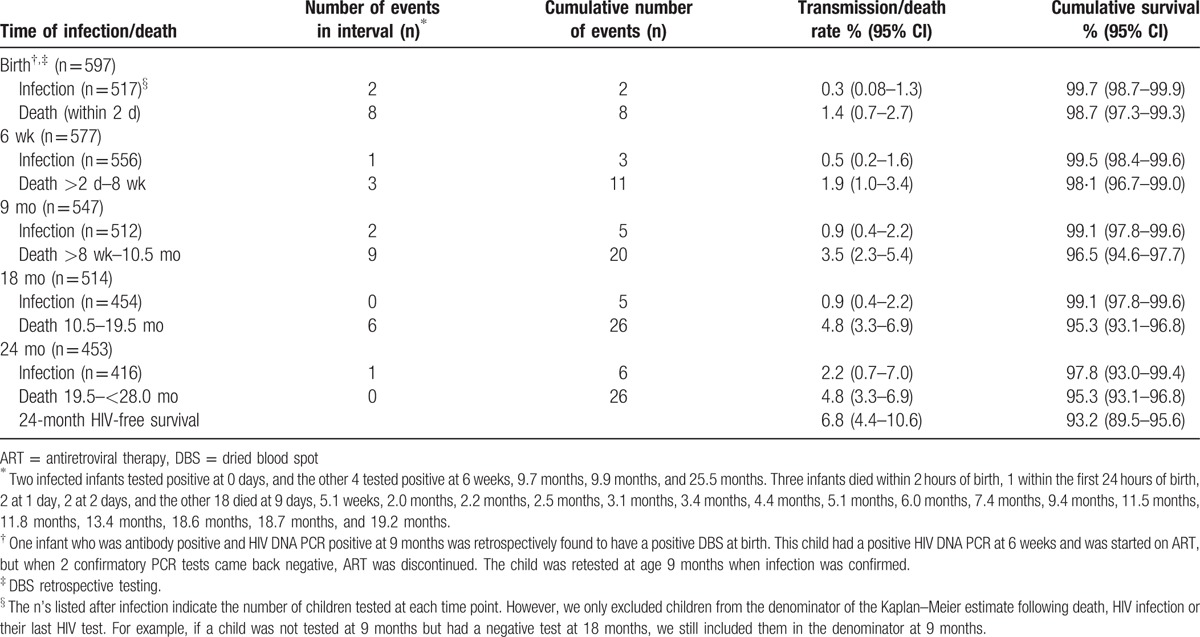
Infant infection status and mortality from Kaplan–Meier estimates.

### 3.3 HIV transmission

The Kaplan–Meier cumulative probability of HIV transmission is shown in Figure 2 and Table 3. A total of 6 HIV-infected infants were identified in the cohort, with an estimated cumulative 24-month transmission rate of 2.2% (95% CI 0.7–7.0). At 6 weeks, 1 infant was confirmed to be HIV-infected with a positive HIV DNA PCR test, for an estimated 6-week cumulative transmission rate of 0.5% (95% CI 0.2–1.6). Testing at 9 months resulted in confirmed HIV infection in 2 additional infants, for an estimated 9-month cumulative transmission rate of 0.9% (95% CI 0.4–2.2). At 18 months, no new HIV infections were identified. At 24 months, 1 additional infant was confirmed as HIV-infected.

DBS specimens collected at birth were retrospectively tested; 2 of the 6 (33.3%) HIV-infected infants had an HIV DNA PCR positive test at birth, reflecting an in utero infection rate of 0.3%. One of the infants with a positive birth HIV DNA PCR was confirmed as HIV-infected at age 9 months. This infant had an initial 6-week positive HIV DNA PCR, and was started on ART pending the confirmatory HIV PCR test result, per Rwandan national guidelines. When 2 subsequent confirmatory test results came back negative, ART was discontinued, and the child was retested at age 9 months, when HIV infection was confirmed. The other infant with a retrospective positive birth PCR was confirmed as HIV-infected following the 6-week test.

Table 4 presents the characteristics of the 6 women who transmitted HIV to their infants, including 1 who reported having had a previous child who was HIV-infected. All 6 transmitting mothers enrolled into the study during pregnancy (third trimester). The duration on ART at delivery for the transmitting women varied from 2.5 to 91.1 months; 3 women had been on ART prior to the current pregnancy, all for >2 years. Only 1 woman reported missing 1 pill when 3-day recall was assessed at enrollment; all other transmitting women reported 100% adherence at enrollment and delivery. Despite reported good ART adherence, 5 of 5 women tested at enrollment had detectable VL, ranging from 577 to 272,000 copies/mL, and 5 of 5 women tested at delivery had detectable VL, ranging from 558 to 290,000 copies/mL. At 18 months, 3 of 5 women tested still had detectable VL (24,500–49,000 copies/mL), and at 24 months, 3 of 5 women tested had VL >1000 copies/mL (7,220–172,000 copies/mL). Postpartum ART regimen changes were noted in 2 of the 6 transmitting women; the other 4 women were consistently on their original tenofovir/lamivudine/efavirenz ART regimen. All 6 women reported exclusive breastfeeding through age 3 months (with range up to 7 months), and any breastfeeding for up to 19 months.

**Table 4:**
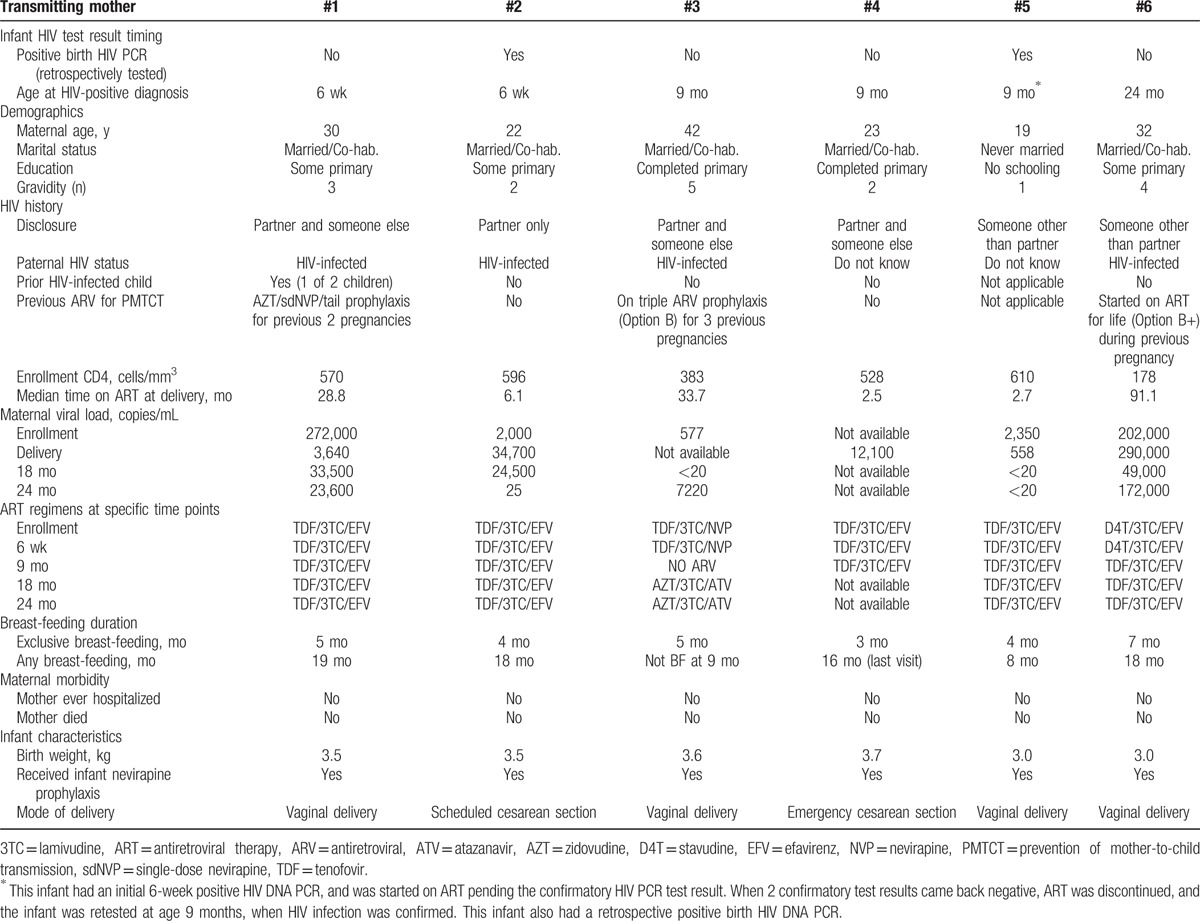
Characteristics of transmitting mothers.

All 6 of the HIV-infected children were initiated on ART with abacavir/lamivudine/lopinavir-ritonavir, and all were alive at 24 months.

### 3.4 HIV-free survival

The Kaplan–Meier cumulative probability of HIV-free survival is shown in Figure 2. Excluding stillbirths, at 6 weeks there were 11 infant deaths and 3 infected infants, with an HIV-free survival of 97.6% (95% CI 96.0–98.6). At 9 months, there were 9 additional infant deaths and 2 additional infected children, resulting in an HIV-free survival of 95.6% (95% CI 93.6–97.0). At 18 months, there were 6 additional deaths, and no additional HIV-infected children, resulting in an HIV-free survival of 94.4% (95% CI 92.1–96.0). At 24 months, there were no additional child deaths, and 1 new infection, for an overall 24-month HIV-free survival of 93.2% (95% CI 89.5–95.6) (Table 3). Of the 415 children testing HIV-negative around 24 months, 11 (2.7%) were still breastfeeding and remained at risk of HIV infection.

## 4 Discussion

The 24-month HIV transmission rate of 2.2% that we report in the Kabeho study is among the lowest, and HIV-free survival rate of 93.2% is among the highest, reported in a real-world clinic setting in breastfeeding HIV-positive women in Africa to date. These results contrast with a household survey conducted in Rwanda in 2009, when ART was given only to pregnant women with CD4 <350 cells/mm^3^, with zidovudine/single-dose nevirapine prophylaxis for those with higher CD4 cell count; transmission at 9 to 24 months was 4.0% with estimated HIV-free survival 91.9%.^[6]^ A survey conducted by the Ministry of Health in July 2015, which sampled sites throughout Rwanda, found a 1.8% mother-to-child transmission (MTCT) rate at 18 months nationally and a 1.3% MTCT rate at 18 months in the 14 Kabeho study sites.^[8]^ The long-term low transmission and high survival rates in the current study occurred despite the health facilities serving women living in significant poverty, with 77% having no education or only attending primary school and few women living in homes with a toilet or piped water, and despite prolonged duration of breastfeeding.

Although low transmission rates have been reported in the context of clinical trials, generally these studies had stopped maternal ART and breastfeeding at 6 months postpartum; for example, in the Botswana Mma Bana trial, 24-month HIV transmission was 1.1%, but median breastfeeding duration was only 5.8 months and ART was provided only through 6 months postpartum in women with CD4 cell count >200 cells/mm^3^.^[9]^ The PROMISE trial is the only clinical trial with provision of maternal ART during prolonged breastfeeding; transmission at age 2 weeks with antepartum ART was 0.6%, similar to our study, and postnatal transmission between 2 weeks and 24 months with postnatal maternal ART was 0.6%, giving a cumulative infection rate of 1.2%.^[10,11]^

Transmission rates in observational studies in clinical settings have generally been higher: in a prospective observational study of 279 breastfeeding HIV-positive women on ART in Zambia, transmission was 4.1% (95% CI 2.2–7.6) at 18 months; in a study of 311 HIV-positive breastfeeding women on ART in Malawi, transmission was 3.2% (95% CI 1.0–5.4) at 24 months; and in a large nationally representative cohort of HIV-exposed infants who were HIV DNA PCR negative at age 4 to 8 weeks and followed prospectively in South Africa when universal maternal ART was recommended, cumulative 18-month transmission was 4.3%.^[12–14]^ Similarly, a recent systematic review of 18 studies evaluating HIV-free survival among breastfed infants of HIV-positive women on ART reported pooled estimates of 12-month and 24-month HIV-free survival of 89.8% (95% CI 86.5–93.2) and 85.8% (95% CI 81.4–90.1), respectively.^[15]^ The substantial proportion of women on ART prior to pregnancy and with suppressed VL may have contributed to the high effectiveness of PMTCT in our study; approximately 64% of women were already receiving ART at the time of their first antenatal care visit. One of the hypothesized benefits of lifelong treatment is protection against transmission in subsequent pregnancies, which is supported by our data. Likewise, in a study of 298 women in Cameroon, in which 51% of women were receiving ART prior to pregnancy, 12-month HIV transmission was 1.2%.^[16]^

However, half of the transmissions occurred in women with long durations of ART (2 to over 7 years). Two out of 3 of these women had a VL >200,000 copies/mL at study enrollment and continued to have high VL throughout the 2-year follow-up period. We had reported previously that HIV-positive pregnant women in the Kabeho study who had received ART >36 months (thus received ART preconception) had higher rates of detectable virus at enrollment compared with women who had been on ART between 4 and 36 months.^[5]^ Other studies have also found that a significant minority of women, who conceived while receiving ART, experienced viral rebound postpartum, which could be associated with increased risk of mother-to-child transmission.^[17]^ These data support the importance of VL monitoring during pregnancy and breastfeeding, and adherence counseling. Rwandan guidelines for management of individuals with VL >1000 copies/mL includes 2 repeat measurements at 3-month intervals, additional adherence counseling and referral for clinical consultation regarding need for second-line therapy if results do not improve, but these may not be consistently implemented.

In our study, 5 of 6 infections (83.3%) occurred by 9 months postpartum; 2 of these were in utero infections, 3 transmissions were due to breastfeeding and the infection at 6 weeks was either the result of intrapartum or early breastfeeding transmission. Most infant deaths (20/26, 76.9%) also occurred by 9 months postpartum. In the South African cohort discussed above, 81% of overall transmission and 56% of postnatal transmission occurred by 6 months postpartum, with 68% of postnatal transmission by 9 months.^[14]^ Thus, the first 6 to 9 months of life appear to be a particularly critical period for follow-up of HIV-exposed infants.

Mortality among live-born HIV-exposed infants in our study was 4.4% (26/597) at 24 months. This is unchanged from a study in Rwanda comparing mortality among HIV-exposed to HIV-unexposed children between 2007 and 2008, when maternal treatment was based on CD4 cell count and women not eligible for treatment received prophylaxis with zidovudine/single-dose nevirapine. In that study, the cumulative risk of death at age 2 years was 4.2% (95% CI 2.2–3.9) among HIV-exposed children compared with 1.5% (95% CI 0.7–1.8) among HIV-nonexposed children.^[18]^ Other studies have reported elevated rates of infant mortality among HIV-exposed but uninfected infants, including those born to mothers who were receiving ART.^[19]^ These data suggest that maternal ART and healthier mothers may not fully eliminate this increased mortality risk in HIV-exposed infants. Further research is needed to better understand the underlying mechanisms for these differences in mortality. Survival of the few known HIV-infected infants in our cohort was excellent; all were rapidly linked to care and initiated on ART, with 100% 24-month survival.

Limitations of our study include residual transmission risk from the 11 infants still breastfeeding following the 24-month HIV test and lack of HIV status among the 10 stillbirths in our cohort, which could decrease our estimate of HIV-free survival. If we assume all stillbirths were HIV-infected, the “worst case” scenario for 24-month HIV-free survival would be 91.6% (95% CI 87.9–94.3). There may have been underreporting of delivery complications and birth defects, as they are based on data from chart review. We also do not have final 24-month transmission outcomes for the 67 mother–infant pairs who were lost to follow-up as well as for those others who withdrew from the study or transferred their care (n = 51). We know at least some of the women who did not present for the 24-month study visit were still attending clinic; if they are considered, the percentage retained after 24 months is nearly 80%. The substantial number of women in this study who defaulted or transferred to another facility highlight the importance of maintaining continuity of care in highly mobile populations. There were also missing data on causes of infant and child death for approximately half of the infants who died. Finally, the Kabeho study includes a representative sample of HIV-positive women and their newborns in urban Kigali only, and only those participating in PMTCT programs in the highest volume sites in Kigali. The sampling method of including in the study all clinics with 50 or more HIV-exposed deliveries a year minimized bias, as women in the study sites had an equal chance of being selected.

In summary, extremely low transmission and high HIV-free survival and few maternal or infant adverse events were observed with implementation of Option B+ in urban Rwanda, which may be due in part to the high proportion of women who were receiving ART prior to the current pregnancy. Our data strongly support the current WHO recommendations for universal ART, and suggest that the goal of eMTCT is possible with universal implementation of treatment for all individuals and with continued vigilance on maintaining viral suppression in those on ART.

## Acknowledgments

The authors thank all of the Kabeho study participants and the staff at study sites for their participation. The authors also recognize all current and former investigators, Kabeho study nurses, the support team at EGPAF, for their contributions to the study well as the Rwanda National Reference Lab and colleagues at USAID for their technical support.
